# Conservative Management of Vaginal Hypoplasia

**DOI:** 10.4274/jcrpe.galenos.2020.2019.S0222

**Published:** 2020-02-06

**Authors:** Özlem Dural, Şükran Poyrazoğlu

**Affiliations:** 1 İstanbul University, İstanbul Faculty of Medicine, Department of Obstetrics and Gynecology, İstanbul, Turkey; 2 İstanbul University, İstanbul Faculty of Medicine, Unit of Pediatric Endocrinology, İstanbul, Turkey

**Keywords:** Adolescent, Müllerian aplasia, androgen-insensitivity syndrome

## Abstract

In patients with Mayer-Rokitansky-Küster-Hauser syndrome and complete androgen insensitivity syndrome (CAIS), management of vaginal hypoplasia includes non-surgical or surgical vaginal elongation techniques. For these patients, primary vaginal dilation is considered a first-line option to avoid the risks of having surgery and complications that may occur due to these procedures. Non-surgical dilation is a highly successful treatment if treatment is initiated when the patient is emotionally mature and ready. Here, we present a case of CAIS with vaginal hypoplasia managed successfully with non-surgical dilation therapy.

## Introduction

 Non-surgical self-dilation treatments should be the primary approach for the management of vaginal hypoplasia in women with Mayer-Rokitansky-Küster-Hauser (MRKH) syndrome and complete androgen insensitivity syndrome (CAIS). Primary vaginal dilation has also been considered as a first-line treatment by the American College of Obstetricians and Gynecologists, because it has a high success rate and has a significantly lower complication risk and much lower cost than surgical vaginoplasty techniques (1,2,3,4,5). Patients should be informed that when they are motivated and ready, most of them (90-96%) will be able to achieve anatomic and functional success with primary vaginal dilation (5). Surgical treatments should be reserved for the rare patient who refuses or fails with dilation therapy. We report an 18 year-old patient with CAIS, who wanted to engage in sexual activity and was referred to the gynecology clinic for the management of vaginal hypoplasia.

## Case Report

A 7 year 2 month old girl was referred to the endocrinology clinic for evaluation of testicle-like masses observed in hernia sacs during a hernioplasty operation. She was born at term with a birth weight of 3600 g, with female external genitalia and was raised as a girl. Her parents were first degree cousins but family history was otherwise unremarkable. There was no family history of disorder of sex diferentiation. Bilateral inguinal hernias were noted at 15 months of age. Histopathological findings of a gonadal biopsy reported immature testis tissue.

On physical examination at her first endocrinology visit, her weight was 26.4 kg [0.7 standard deviation scores (SDS)], and height was 126.2 cm (0.8 SDS). Physical examination was normal. Gonads were palpable in the inguinal canals bilaterally. Pubic hair and breast development were both at Tanner stage 1. No virilization symptoms were observed.

Karyotype analysis was 46,XY. Hormone analysis at the age of 21 months showed elevated luteinizing hormone and normal follicle stimulating hormone, estradiol and testosterone concentrations. She had normal testosterone response to human chorionic gonadotropin (hCG) stimulation test. Anti-Müllerian hormone was also within the normal range for male infants (Table 1). Ultrasonography of the pelvis showed no uterus, fallopian tubes, or ovaries. Based on her physical exam and biochemical findings, she was assigned a clinical diagnosis of CAIS. Genetic analysis of the androgen receptor gene was performed, and a hemizygous mutation (c.2668G>A, p.Val890Met) was identified in exon 8.

She underwent regular tumor surveillance throughout childhood, including physical examinations, measurement of α-fetoprotein and hCG, and testicular ultrasound examinations. Breast development started (Tanner stage 2) at age 9 years 10 months, while pubic hair was at Tanner stage 1.

At age 14 years 8 months, her weight was 47.3 kg (-1.2 SDS), height was 160 cm (-0.2 SDS). Physical examination revealed normal female external genitalia and breast development (Tanner stage 5) with sparse pubic hair (Tanner stage 2) and axillary hair. Bilateral gonadectomy was performed at age 14 years 9 months due to the presence of inguinal testes and increased potential risk for the development of a germ cell tumor. Histological analysis of the excised gonads revealed that they consisted of testicular tissue, characterized by immature seminiferous tubules. Estrogen replacement therapy was initiated with transdermal 17 b estradiaol starting at a dose of 50 µg and increasing to 100 µg over a period of one year.

At the age of 18 years she was referred to the outpatient pediatric gynecology clinic for the management of vaginal hypoplasia because she wanted to become sexually active. Physical examination revealed normal breast development, normal female external genitalia, an unformed hymen and blind vaginal pouch with a length of about 3 cm. Pubic hair and breast development were at Tanner stage 2 and 5, respectively.

Since she was emotionally and physically ready to begin primary vaginal dilation treatment, her external genital anatomy was reviewed with the patient, following a detailed explanation of all the stages of the treatment. She was instructed how to use Pyrex tubes of gradually increasing size and diameter and seen in clinic every two weeks. Anatomical success with a 6 cm long vagina was achieved after a clinical follow-up of approximately two months. No complications or adverse effects were encountered during the treatment. She was informed that she would need to continue dilation 2-3 times per week until she began to have regular penetrative intercourse. We also reviewed the methods to decrease sexually transmitted infections, including the use of condoms and the human papilloma virus (HPV) vaccine was recommended.

## Discussion

Frank’s Method involves the use of vaginal dilators, which increase in size and diameter over time, to stretch a short vagina to a larger length and diameter (6). Although it is a highly effective therapy, many reproductive health providers have little experience of how to guide patients through this process. Dilator therapy should only be suggested to women with high motivation, for example those in a current relationship or those who want to engage in sexual activity. Patients over 18 years of age at the start of treatment have significantly higher anatomic success rates, possibly related to motivational reasons (1). It should be noted that initial vaginal length, even when the vagina appears as a dimple below the urethra, is not associated with anatomical or functional success, but may be associated with duration of dilator therapy. Although there is no difference in anatomic success rates, patients with CAIS have a larger starting vaginal length than patients with MRKH syndrome (7).

Initially, a thorough examination of the external genital anatomy of the patient should be performed with the aid of a mirror and the patient should be able to understand and show the appropriate location and angle for inserting the dilator. She should be instructed to place a small dilator on the distal vaginal apex in a downward angle 1-3 times per day for 10-30 minutes. The patient should be followed up at the clinic at least every two weeks and the length and width of the dilators given should be progressively increased. There is no vaginal length or width specified to begin penetrative intercourse as even elongation by gentle vaginal intercourse alone can be successful with a supportive partner (8,9). Although success has been described anatomically by obtaining a vagina of 6 cm or longer (1), the best definition of success is a functional vagina that is sufficient for comfortable sexual activity reported by the patient (3). The most common adverse effects with primary vaginal dilation are bleeding, pain and urinary symptoms. Increasing use of lubricants and discontinuing treatment until bleeding ceases are the most commonly used approaches in the management of these complications (5).

In our case, an 18 year-old patient with CAIS, who wanted to engage in sexual activity, was referred to the gynecology clinic for the management of vaginal hypoplasia. Anatomical success with a 6 cm long vagina was achieved in two months and no complications or adverse effects were encountered. During the treatment, methods to decrease sexually transmitted infections, including the use of condoms were also reviewed and HPV vaccine was recommended, since patients with MRKH syndrome and CAIS are at risk of vulvovaginal HPV infection (10).

For patients with poor adherence or failure to succeed with dilation therapy or for those who do not wish to start vaginal dilation therapy, many surgical vaginoplasty methods have been described, including the Vecchietti procedure, McIndoe procedure and sigmoid vaginoplasty. In the literature, there is no consensus on the surgical technique to be chosen to achieve the best functional outcome and sexual satisfaction (11). Complications of these operations include injuries of adjacent organs, such as bladder or rectum, graft necrosis, neovaginal granulation tissue and fistulae. In the long term, all surgical methods carry a risk for vaginal stenosis or strictures, and long-term reoperation rates might be as high as 40% (1,12). Therefore, most procedures also require ongoing postoperative dilation to decrease the risk of stenosis.

## Figures and Tables

**Table 1:**
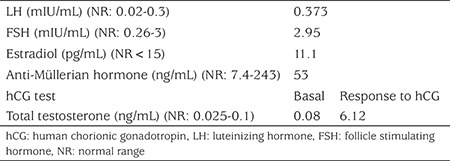
Hormone analysis of the patient

## References

[1] Callens N, De Cuypere G, De Sutter P, Monstrey S, Weyers S, Hoebeke P, Cools M (2014). An update on surgical and nonsurgical treatments for vaginal hypoplasia. Hum Reprod Update.

[2] Willemsen W, Kluivers KB (2015). Long-term results of vaginal construction withthe use of Frank dilation and a peritoneal graft (Davydov procedure) in patients with Mayer-Rokitansky-Ku¨ ster syndrome. Fertil Steril.

[3] Edmonds DK, Rose GL, Lipton MG, Quek J (2012). Mayer–Rokitansky– Kuster–Hauser syndrome: a review of 245 consecutive cases managed by a multidisciplinary approach with vaginal dilators. Fertil Steril.

[4] Routh JC, Laufer MR, Cannon GM Jr, Diamond DA, Gargollo PC (2010). Management strategies for Mayer–Rokitansky–Küster–Hauser related vaginal agenesis: a cost-effectiveness analysis. J Urol.

[5] No Authors List (2018). Committee on Adolescent Health Care. ACOG Committee opinion no. 728: Mullerian agenesis: diagnosis, management, and treatment. Obstet Gynecol.

[6] Frank R (1938). The formation of an artificial vagina without operation. Am J Obstet Gynecol.

[7] Ismail-Pratt IS, Bikoo M, Liao LM, Conway GS, Creighton SM (2007). Normalization of the vagina by dilator treatment alone in Complete Androgen Insensitivity syndrome and Mayer-Rokitansky-Kuster- Hauser syndrome. Hum Reprod.

[8] Moen MH (2014). Vaginal agenesis treated by coital dilatation in 20 patients. Int J Gynaecol Obstet.

[9] Callens N, Weyers S, Monstrey S, Stockman S, van Hoorde B, van Hoecke E, De Cuypere G, Hoebeke P, Cools M (2014). Vaginal dilation treatment in women with vaginal hypoplasia: a prospective one-year follow-up study. Am J Obstet Gynecol.

[10] Frega A, Scirpa P, Sopracordevole F, Biamonti A, Bianchi P, De Sanctis L, Lorenzon L, Pacchiarotti A, French D, Moscarini M (2011). Impact of human papillomavirus infection on the neovaginal and vulvar tissues of women who underwent surgical treatment for Mayer-Rokitansky- Kuster-Hauser syndrome. Fertil Steril.

[11] Laufer MR (2002). Congenital absence of the vagina: in search of the perfect solution. When, and by what technique should a vagina be created?. Obstet Gynecol.

[12] Cheikhelard A, Bidet M, Baptiste A, Viaud M, Fagot C, Khen-Dunlop N, Louis-Sylvestre C, Sarnacki S, Touraine P, Elie C, Aigrain Y, Polak M;, French MRKH Study Group: (2018). Surgery is not superior to dilation for the management of vaginal agenesis in Mayer-Rokitansky-Kuster-Hauser syndrome: a multicenter comparative observational study in 131 patients. Am J Obstet Gynecol.

